# Emergency department physician training in Jamaica: a national public hospital survey

**DOI:** 10.1186/1471-227X-8-11

**Published:** 2008-10-12

**Authors:** Ivor W Crandon, Hyacinth E Harding, Shamir O Cawich, Eric W Williams, Jean Williams-Johnson

**Affiliations:** 1 The Department of Surgery, Radiology, Anaesthesia and Intensive Care. The University Hospital of the West Indies, Mona, Kingston 7, Jamaica, West Indies; 2 The Department of Basic Medical Sciences. The University of the West Indies, Mona, Kingston 7, Jamaica, West Indies; 3 Emergency Medicine Division, Department of Surgery, Radiology, Anaesthesia and Intensive Care. The University of the West Indies, Mona, Kingston 7, Jamaica, West Indies

## Abstract

**Background:**

Emergency Department (ED) medical officers are often the first medical responders to emergencies in Jamaica because pre-hospital emergency response services are not universally available. Over the past decade, several new ED training opportunities have been introduced locally. Their precise impact on the health care system in Jamaica has not yet been evaluated. We sought to determine the level of training, qualifications and experience of medical officers employed in public hospital EDs across the nation.

**Methods:**

A database of all medical officers employed in public hospital EDs was created from records maintained by the Ministry of Health in Jamaica. A specially designed questionnaire was administered to all medical officers in this database. Data was analyzed using SPSS Version 10.0.

**Results:**

There were 160 ED medical officers across Jamaica, of which 47.5% were males and the mean age was 32.3 years (SD +/- 7.1; Range 23–57). These physicians were employed in the EDs for a mean of 2.2 years (SD +/- 2.5; Range 0–15; Median 2.5) and were recent graduates of medical schools (Mean 5.1; SD +/- 5.9; Median 3 years).

Only 5.5% of the medical officers had specialist qualifications (grade III/IV), 12.8% were grade II medical officers and 80.5% were grade I house officers or interns. The majority of medical officers had no additional training qualifications: 20.9% were exposed to post-graduate training, 27.9% had current ACLS certification and 10.3% had current ATLS certification.

**Conclusion:**

The majority of medical officers in public hospital EDs across Jamaica are relatively inexperienced and inadequately trained. Consultant supervision is not available in most public hospital EDs. With the injury epidemic that exists in Jamaica, it is logical that increased training opportunities and resources are required to meet the needs of the population.

## Background

Jamaica is a developing Caribbean country that has an estimated population of 2.67 million persons [1]. Emergency health needs are provided by a complement of 23 public hospitals across the island [2,3]. Pre-hospital emergency response services, although present, are not universally available throughout the island [4-8]. Most patients requiring emergency care present to hospital after transport by non-medical personnel without proper resuscitation [7-9]. Therefore medical officers in the Emergency Department (ED) are often the first medical responders to acute emergencies in this setting.

One of the requirements for the optimal provision of emergency medical care is for the ED to be staffed by physicians who can assess and treat patients in a timely fashion and competently make rapid critical decisions. This requires a strong knowledge base, additional training and experience. Nevertheless, there is wide variation in the level of experience and qualifications of medical officers in EDs throughout Jamaica because there are no specific requirements for employment beyond a valid general medical degree.

Over the past decade, several initiatives have resulted in the introduction of new training opportunities aimed at improving the calibre of medical officers staffing the EDs. These include the introduction of training programmes in Basic Cardiac Life Support (BLS) and Advanced Cardiac Life Support (ACLS) in 1998 as well as the Advanced Trauma Life Support (ATLS) training programme in 2001 [2]. Additionally, the University of the West Indies (UWI) commenced a four year post-graduate training program in Emergency Medicine in 1997 [2].

These developments have resulted in an increased number of trained ED staff at certificate, diploma and degree levels. Some of these physicians are now employed in EDs throughout Jamaica. The precise impact of these available training opportunities on the health system in Jamaica has not yet been evaluated. We sought to determine the level of training, qualifications and experience of the medical officers in public hospital EDs across the nation.

## Methods

In Jamaica, the Ministry of health (MOH) recruits all medical officers in public hospitals without assistance from external recruiting agencies. The MOH maintains complete records of currently employed physicians across the nation. These data were accessed to compile a database on the ED staff complement of all public hospitals across Jamaica.

Public hospitals are classified by the MOH [10] according to the level of service provided and the population served (Table 1). Medical officers were classified by the MOH [10] according to their qualification and the number of years after graduation from medical school (Table 2). This grading system was used as an indicator of the level of experience of medical officers.

**Table 1:** Public hospital type as designated by the ministry of health in jamaica

**Type**	**Level of Care**	**Services offered**
Specialist	Multi-disciplinary care	Type A hospital with specialized patient population
Type A	Multi-disciplinary care	Tertiary referral hospital with access to sub-speciality care and advanced investigational facilities
Type B	Large Urban hospital	Secondary care in basic specialties: general surgery, paediatrics, internal medicine, obstetrics and gynaecology.
Type C	Basic district hospital	Inpatient and outpatient care with basic radiographic and laboratory services

Taken from: Jamaica Ministry of Health. Medical Staff Manual. 1995 ^(10)^

**Table 2:** Medical officer grade as designated by ministry of health in jamaica

**Grade**	**Post-graduate degree**	**Registration**	**Years post graduation from medical school**
1	None	Provisional/full	< 2
2	None	Full registration	> 2
3	Yes	Full registration	> 5 but not employed in consultant post
4	Yes	Full registration	> 5 and employed in a consultant post

Taken from: Jamaica Ministry of Health. Medical Staff Manual. 1995 ^(10)^

A questionnaire was designed as the instrument to collect information from the medical officers in the database. The questionnaire was not pre tested. It sought information from the medical officers regarding their training, qualifications and experience. Approval was obtained from the local ethics committee at the MOH/UWI to access their records and perform the questionnaire study. Permission to administer the questionnaire was secured from the Senior Medical Officer of each hospital. The medical officers were then individually contacted and verbal consent to administer the questionnaire was secured. The questionnaires were completed by telephone interview for rural hospitals and by direct interview in urban hospitals. Data from incomplete questionnaires were not included in the final analysis.

Data were analyzed using the Statistical Package for the Social Sciences (SPSS) version 10.0. Data were expressed as frequencies or means with standard deviations as appropriate.

## Results

There were 23 public hospitals across the island of Jamaica. Two specialist hospitals were identified: the Bustamante Children's Hospital providing tertiary care only to children under the age of 12 years and the National Chest Hospital providing tertiary care to adults with respiratory diseases. There were three type A hospitals: The Cornwall Regional Hospital, the Kingston Public Hospital and the University Hospital of the West Indies. The remaining institutions were type B (4) and C (14) hospitals.

There were 161 posts available for ED medical officers across these 23 hospitals, of which 160 posts were occupied. Of these, 71 (44.1%) posts were clustered in hospitals in the capital city, Kingston. The medical officers had full time appointments in the ED in 97 (60.2%) cases and part-time ED appointments in 64 (39.8%) cases.

**Figure 1:**
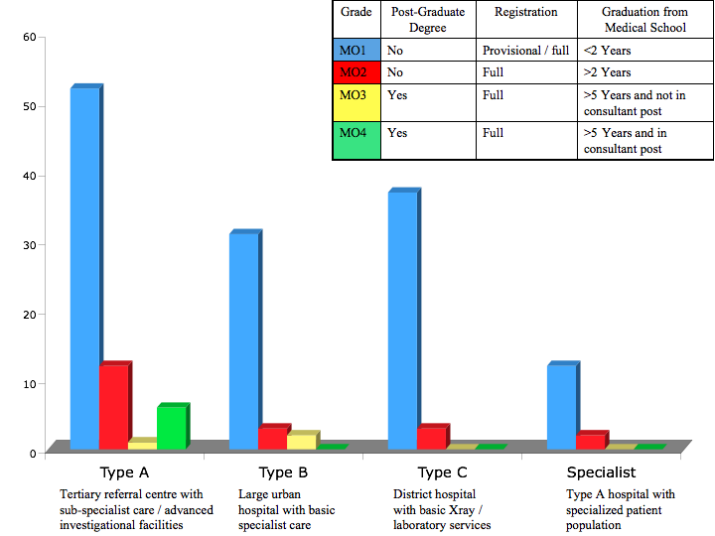
**The distribution of medical officers according to their employment grade and hospital category**.

The distribution of medical officers according to their employment grade and hospital category is outlined in Figure 1. It was notable that only 6 (3.7%) posts were available for consultant (Grade IV) grade staff. The majority of posts (132/160, 82%) were appointed to Grade I medical officers who were not career emergency physicians.

A single investigator administered questionnaires to 160 ED medical officers. Of this, 146 (90.7%) of questionnaires were completed. The demographics of the surveyed medical officers are outlined in Table 3.

**Table 3:** Demographics of emergency department medical officers across public hospitals in jamaica (n = 146)

**Characteristic**	**Number (%)**
Mean age in years	32.3 (SD ± 7.1; Range = 23–57)
Male Gender	69 (47.3%)
Mean Interval since medical school graduation in years	5.1 (SD ± 5.9; Median 3.0)
UWI Graduate	94 (64.3%)
Full Time Appointment in the ED	97 (60.2%)
Commenced postgraduate training	30 (20.6%)
Mean Duration of ED Appointment in years	2.21 (SD = /-2.5; Range = 0–15)

The medical officers were employed in the ED for a mean of 2.2 years (SD +/- 2.5; range 0–15). In terms of experience, 30 (20.6%) medical officers had commenced post-graduate training and only 7 (4.8%) of them completed residencies in emergency medicine. The respondents had graduated from medical school for a mean of 5.1 years (SD +/- 5.9; median 3; range 1–34).

There were 113/146 responders for ACLS training. Only 43 (38.1%) of these 113 responders had certification in ACLS and only 31 (27.9%) had current ACLS certification.

There were 114/146 medical officers who completed responses to ATLS training. Of these, 37 (32.5%) had completed ATLS training courses and only 15/146 (10.3%) had valid certificates.

## Discussion

The ED is often the first line of contact for trauma patients and those seeking care for acute emergencies. Over the past decade there have been significant advancements in the provision of emergent care in EDs across Jamaica [2]. Their impact on the delivery of emergent care in Jamaica has not been formally assessed.

One of the most notable developments has been the inception of a four year Emergency Medicine post-graduate training program through the UWI in 1997 [2]. Up to July 2006, there have been 12 graduates of the specialty program [2]. Some of these specialists have been appointed to grade IV consultant posts at EDs across Jamaica.

It has been suggested that consultant supervision in EDs will improve the quality of emergent patient care [2,11] and reduce the number of preventable deaths [12,13]. While there is no agreed optimal consultant to non-specialist ratio, many advisory bodies have made recommendations unique to their territories. An Australasian recommendation is for at least 1 consultant per shift in the highest level EDs, while smaller hospitals with 150 beds or less (Type B/C equivalent) should have at least 2 consultants available per day [11]. A more demanding recommendation has been proposed in Britain where each unit should employ 8 consultants to cover all shifts [14].

This survey has revealed that only 3.7% of all available posts in EDs across Jamaica are grade IV consultant posts. This leaves many public hospitals in Jamaica without any consultant supervision in the ED. In fact, none of the specialist hospitals in Jamaica are provided with any posts for Grade IV consultant appointments.

This is strikingly different to the situation in other countries. In the nearest Caribbean country, the Cayman Islands, the major tertiary referral (Type A equivalent) hospital houses an 11 bed ED that employs 3 emergency medicine specialists (grade IV equivalent), accounting for 33% of the ED staff complement [15]. This makes at least one specialist available per shift 24 hours a day. This is similar to reports from other countries that have documented 32% of their staff complement being the equivalent of consultant/grade IV medical officers [16].

At present, graduates of the UWI training program occupy all 6 consultant posts and 2 grade III posts. This number of available posts is grossly inadequate considering that there are 23 hospital EDs across Jamaica.

A major limitation is the assumption that staffing public hospital EDs with graduates of local training programmes will result in improved patient care. Nevertheless, we believe it is reasonable to propose that all Type A, B and Specialist hospitals should have some consultant supervision in their EDs. We believe that all Type A and Specialist hospitals should be afforded a minimum of 4 consultant posts to ensure at least 1 consultant per 8 hour shift. Additionally, each of the 4 Type B hospitals should have a minimum of 3 consultant posts. This would mean that there should be a minimum of 32 consultant posts available across the EDs in Jamaica.

This situation demands desperate attention by policy makers to increase the number of available grade III and IV posts in public hospital EDs. This would prevent emigration of qualified Emergency Medicine specialists and increase the attractiveness of the UWI post-graduate training programme to young physicians who may be deterred by the lack of available employment opportunities after graduation.

Junior level medical officers without specialized Emergency Medicine training make up the bulk of the ED personnel in many hospitals across the hemisphere [17]. However, many of these institutions stipulate that medical officers receive some form of additional training prior to employment in this setting. Certification in ATLS and ACLS are common requisites [17,18]. We believe it is reasonable to propose that all medical officers should have ACLS/ATLS certification prior to employment in Jamaican EDs.

Many opportunities for appropriate training exist in Jamaica. Certification in ATLS was introduced through the UWI in May 2001 [2]. The Jamaican chapter of the American College of Surgeons conducts 3 courses per year, providing certification for up to 75 persons annually [2,12]. Similarly, the Heart Foundation of Jamaica in conjunction with the MOH conducts several courses for ACLS certification in Jamaica since April 1998 [2].

Our survey has shown that grade I medical officers make up 82% of the physician complement within public hospital EDs across Jamaica. Unfortunately, many of these medical officers are relatively inexperienced, with current ACLS certification in 27.9% and current ATLS certification in 10.3% of respondents. Even in those with prior certification, revalidation rates were low. This situation is not unique to Jamaica [19] and may require specific targeting and policy enforcement for compliance.

Many of the medical officers who were not ACLS/ATLS certified had little practical experience since most were employed in the ED for a mean of 2.2 years and were recent graduates of medical school within a mean of 5.1 years. Prerequisites for ACLS/ATLS certification with policy enforcement is one way to ensure that these relatively inexperienced medical officers gain additional training prior to employment in the ED. In our circumstances, we must also consider employment incentives for non-specialist medical officers with appropriate training in EDs, as is done in other countries [16,17,19].

It was notable that all graduates of the UWI Emergency Medicine programme practicing in grade III/IV posts in Jamaica were employed in Kingston. There were no grade III/IV officers practicing in rural areas to offer specialist supervision in the public hospitals EDs outside of Kingston. This is similar to the situation in other developed countries [20,21] and has been linked to the lack of sophisticated support services, inadequate professional development opportunities and an insufficient population size to sustain professional interest. This is a difficult problem to overcome since these EDs need competent medical officers to supervise and deliver care. We may learn from models used in other countries where general practitioners with additional training [21-23] or experienced nurse practitioners [24,25] have effectively staffed EDs with exemplary success.

## Conclusion

The public hospital EDs across Jamaica are staffed by a preponderance of junior medical officers with little emergency medicine experience, low ACLS/ATLS certification rates and little post-graduate training. Mandatory certification for ED medical officers with policy enforcement may be necessary to increase the level of training.

Additionally, policy makers must increase the number of available consultant posts available in these public hospitals to ensure the highest possible standard of clinical care. Particularly with the injury epidemic that exists in Jamaica and the present state of training and experience of A&E officers, it is logical that increased training opportunities and resources are required to optimally meet the needs of the population.

## Competing interests

The authors declare that they have no competing interests.

## Authors' contributions

IC conceived the study, participated in its design and drafted the manuscript. HH participated in the study design, statistical analysis and drafting of the manuscript. EW participated in data acquisition, and helped to draft the manuscript. SC participated in data acquisition, study design and drafting of the manuscript. JWJ participated in data acquisition and helped in drafting the manuscript. All authors have read and approved the final manuscript.

## Pre-publication history

The pre-publication history for this paper can be accessed here:


